# Structural Engineering of Anode Materials for Low-Temperature Lithium-Ion Batteries: Mechanisms, Strategies, and Prospects

**DOI:** 10.1007/s40820-024-01363-y

**Published:** 2024-03-11

**Authors:** Guan Wang, Guixin Wang, Linfeng Fei, Lina Zhao, Haitao Zhang

**Affiliations:** 1 Beijing Key Laboratory of Ionic Liquids Clean Process, Institute of Process Engineering, Chinese Academy of Sciences, Beijing, 100190 People’s Republic of China; 2 School of Chemical Engineering, Sichuan University, Chengdu, 610065 People’s Republic of China; 3 School of Energy Materials and Chemical Engineering, Hefei University, Hefei, 230601 People’s Republic of China; 4 School of Materials Science and Engineering, Nanchang University, Nanchang, 330031 People’s Republic of China; 5 Key Laboratory of Polymer and Catalyst Synthesis Technology of Liaoning Province, School of Environmental and Chemical Engineering, Shenyang University of Technology, Shenyang, 110870 People’s Republic of China

**Keywords:** Low-temperature performance, Anode materials, Microstructural regulations, Surface modifications

## Abstract

The severe degradation of electrochemical performance for lithium-ion batteries (LIBs) at low temperatures poses a significant challenge to their practical applications. Consequently, extensive efforts have been contributed to explore novel anode materials with high electronic conductivity and rapid Li^+^ diffusion kinetics for achieving favorable low-temperature performance of LIBs. Herein, we try to review the recent reports on the synthesis and characterizations of low-temperature anode materials. First, we summarize the underlying mechanisms responsible for the performance degradation of anode materials at subzero temperatures. Second, detailed discussions concerning the key pathways (boosting electronic conductivity, enhancing Li^+^ diffusion kinetics, and inhibiting lithium dendrite) for improving the low-temperature performance of anode materials are presented. Third, several commonly used low-temperature anode materials are briefly introduced. Fourth, recent progress in the engineering of these low-temperature anode materials is summarized in terms of structural design, morphology control, surface & interface modifications, and multiphase materials. Finally, the challenges that remain to be solved in the field of low-temperature anode materials are discussed. This review was organized to offer valuable insights and guidance for next-generation LIBs with excellent low-temperature electrochemical performance.

## Highlights

- The working principles and limitations of current anode materials at low temperatures are elucidated.
- Advantages and emphases of various modification strategies, including structural design, morphology control, surface & interface modifications, and multiphase materials of low-temperature anode materials, are reviewed.
- Perspectives and challenges in developing novel low-temperature anode materials are discussed.

## Graphical abstract

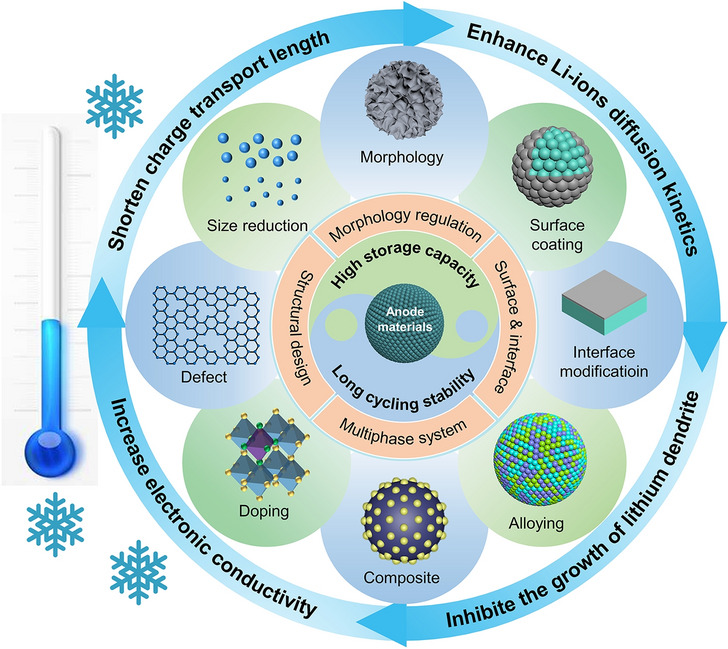

## Introduction

Accompanied with the expeditious transition toward green energy and the global consensus on carbon neutrality, lithium-ion batteries (LIBs) have emerged as the primary energy storage devices in a wide range of applications due to their exceptional merits, including high energy density and long operational lifespan [1–3]. For instance, electric vehicles (EVs) powered by LIBs have attracted substantial attention in the past decade [4]; as estimated by the International Energy Agency, the global EVs fleet was poised to reach an astonishing 230 million by 2030 [5]. Furthermore, LIBs are progressively broadening their application horizons, aiming to supplant conventional nickel–cadmium batteries in specialized domains such as polar research, deep-sea detection, military installation, and space exploration [6]. In accordance with these complex application scenarios [7], LIBs are expected to be capable of withstanding temperatures as low as − 40 °C in civilian applications; moreover, LIBs are required to function at lower temperatures, which is about − 60 °C for military operations and polar expeditions, and even below − 80 °C for deep-sea detection and space exploration [8]. However, the electrochemical performance of state-of-the-art LIBs exhibits a significant decline under − 40 °C, normally maintaining an energy density of merely 5% and power density of 1.25% compared to those at room temperature (RT) [9]. Consequently, the development of LIBs with excellent low-temperature (LT) performance has become an urgent task.

**Fig. 1:**
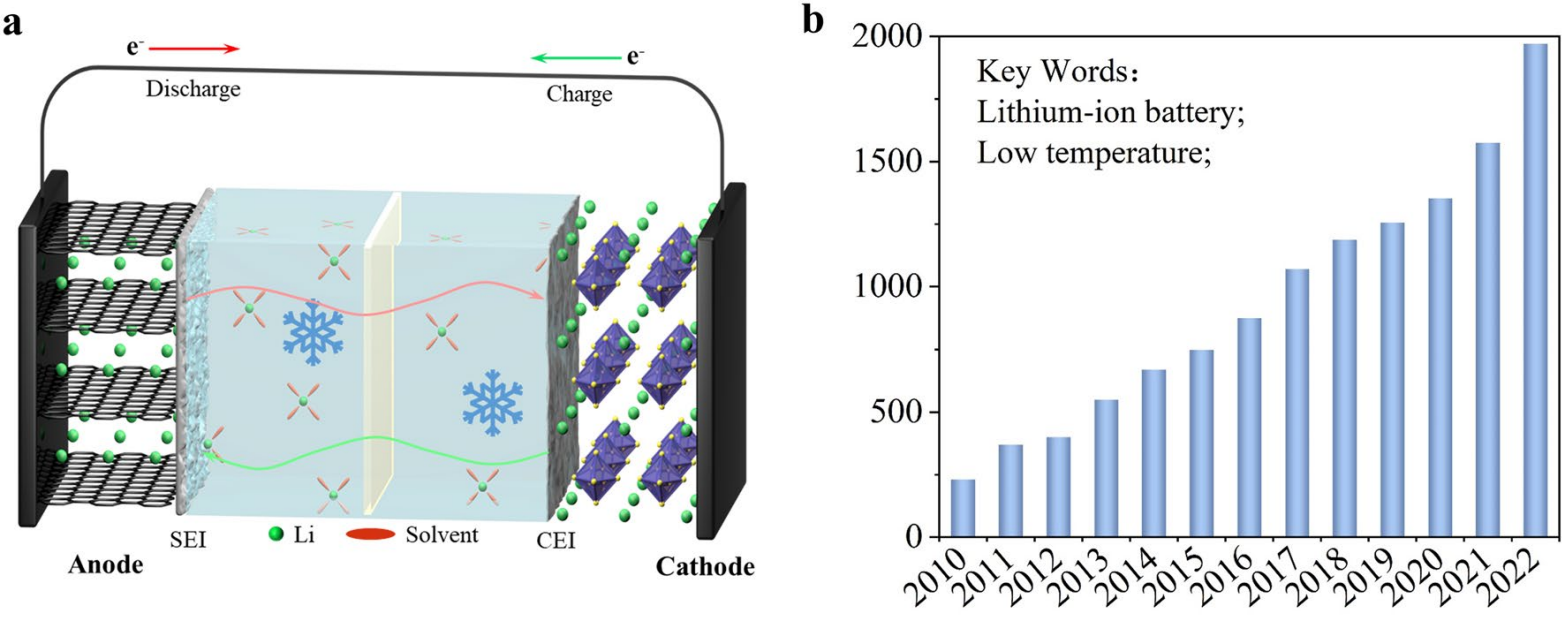
** a** Schematic for the LT-induced issues of LIBs. **b** Numbers of published articles related to LT LIBs during 2010–2022 (data from Web of Science)

The typical structure of LIBs (Fig. 1a) unveils the multiple issues that can impact their performance at LT [10–17]. Firstly, the viscosity of liquid electrolyte experiences a sharp escalation and even solidification, resulting in a decrease in wettability and ionic conductivity. Then, the Li^+^ (de)solvation processes become sluggish, thereby severely impeding the insertion/extraction of Li^+^ in electrodes, which leads to a significantly augmented charge-transfer resistance (*R*_ct_) and diminished Li^+^ diffusion kinetics. Thirdly, the increased bulk resistance and depressed ion diffusion within electrodes inhibit the intercalation and deintercalation processes, which induces a significantly declined capacity. Finally, the polarization-induced formation of lithium dendrites may get even worse at LT and cause additional safety hazards. These aforementioned restrictions jointly contribute to a severe deterioration of electrochemical performance for LIBs in LT environments, and specially, it is important to note that the realization of fast Li ion diffusion within the anode is widely acknowledged as a bottleneck in improving the LT performance of LIBs [18–20], which is the central topic of this current review.

In this context, it is not surprising that significant amount of papers have been published regarding LT LIBs in past years (Fig. 1b), and solid achievements on the improvement of LT performance for LIBs through either judicious selection of suitable electrolytes or novel design of electrode materials have been demonstrated [21–23]. Accordingly, there are also a few seminal reviews focusing on the advancements in LT electrode materials [24, 25]. However, in this review, we will concentrate on the crucial structural characteristics, which are essential for improving the LT performance of anode materials; furthermore, we will also attempt to systematically illustrate the working mechanisms behind each modification strategy; therefore, this review is anticipated to provide valuable insights for the design and applications of LT anode materials.

Our review will be organized as follows. First, we will briefly introduce the present understanding of working principles for LT anodes. Second, several commonly used LT anode materials as well as their advantages and disadvantages will be stated. Third, we will emphasize the latest developments in LT anode materials, with our special attention aimed at modification strategies of these materials that can effectively improve their LT performance. Four kinds of modification strategies, which are structural design, morphology control, surface and interface modifications, and multiphase system, will be discussed. We will also extract the mechanisms behind each modification strategy and how they contribute to improving the performance of low-temperature anode materials. Finally, we will try to give our outlook on the challenges for developing practicable LT anodes.

## Key Parameters Concerning Anode Materials

To develop high-performance LT anode materials, it is essential to understand the influences of LT on the electrochemical reaction process. As discussed above, the electrochemical reactions concerning the operations of LIBs would be severely inhibited at LT. Generally, the relationship between temperature and electrochemical reaction rate can be described by the Arrhenius equation [26]:

1 $$\begin{array}{*{20}c} {\kappa = Ae^{{ - \frac{{E_{{\text{a}}} }}{RT}}} } \\ \end{array}$$

Here, $\kappa$ represents the electrochemical reaction rate within LIBs, *A* is the pre-exponential factor, $E_{{\text{a}}}$ denotes the activation energy, *T* signifies the absolute temperature, and *R* symbolizes the ideal gas constant. Principally, the reaction rate experiences an exponential decline as the temperature decreases [27]; thus, it becomes difficult to achieve an expeditious reaction rate at LT. For example, the diffusion of Li^+^ and the transfer of electrons within the architecture of anode materials are largely limited at LT, and these rates are greatly dependent on the intrinsic electronic and ionic conductivities of electrodes [18]. Therefore, the rates of electron transfer and Li^+^ diffusivity inside electrode materials are the key parameters for the batteries’ LT performance. Besides, Li deposition on the surface of anodes at LT constitutes another noteworthy issue, because it engenders performance degradation and safety concerns [28].

### Electronic Conductivity

According to the electron-sea model [29], the valence band of electrode material motivates and facilitates the transfer of analog electrons into the conduction band, thereby enabling the conduction of electricity; the primary determinant of electron conductivity lies in electron mobility ($\sigma$), which represents the ability of electrons to move freely within the material [13]:

2 $$\begin{array}{*{20}c} {\sigma = n_{i} e\mu_{{\text{e}}} + p_{i} e\mu_{{\text{h}}} } \\ \end{array}$$

Here, *n*_*i*_ is the concentration of electrons, *μ*_e_ represents the mobility of electrons, *p*_*i*_ signifies the concentration of holes, *μ*_h_ corresponds to the hole mobility, and *e* signifies the absolute valence state of the charge carrier. To achieve reasonable LT performance, anode materials should possess a high intrinsic electronic conductivity. The intrinsic electron conductivity of most materials is mainly determined by their crystal structure and interfacial characteristics [30], such as excellent LT anode materials, compounds range from graphite to metallic materials (with small band gaps). So far, many approaches have been proposed to enhance the LT electronic conductivities of anode materials by modifying their microstructures and interfaces (defects engineering, heteroatomic doping, surface coating, etc.). Specifically, the introduction of defects or doping can effectively induce additional charge carriers, resulting in enhanced electron conductivity for the material [31, 32]. Additionally, the application of conductive coatings on material surfaces can facilitate the transfer of electrons, reduce the interface impedance, and provide excellent chemical stability, resulting in improved LT performance.

### Ion Diffusivity

Ion diffusion coefficient ($D_{i}$, also called diffusivity) is another important parameter to characterize anode materials, which quantifies the migration rate of lithium ions within materials. The diffusion process can be described by Arrhenius Eq. 3 [12], and the diffusion time (*t*) of Li^+^ can be calculated through Eq. 4:

3 $$\begin{array}{*{20}c} {D_{i} = D_{0} e^{{ - \frac{\Delta G}{{K_{{\text{B}}} T}}}} } \\ \end{array}$$

4 $$\begin{array}{*{20}c} {t = \frac{{x^{2} }}{{qD_{i} }}} \\ \end{array}$$

Here, $D_{0}$ signifies the estimated pre-exponential factor, $K_{{\text{B}}}$ represents the Boltzmann constant, ${\Delta }G$ denotes the change of Gibbs free energy, *x* is the diffusion distance of Li^+^ within electrode materials, and *q* represents the constant of dimensionality. Specifically, the values of *q* are assigned as 2, 4, and 6 for materials with one-dimensional (1D), two-dimensional (2D), and three-dimensional (3D) characteristics, respectively. Generally, $D_{i}$ exhibits an exponential recession as the temperature decreases, leading to a suppressed LT electrochemical performance for electrode materials. Intuitively, a promising approach to address this issue involves the search of novel materials with reduced diffusion barriers (${\Delta }G$) [24]. For existing electrode materials, it is feasible to achieve shortened diffusion times (*t*) by modulating their morphological and microstructural features [33]; specifically, the diffusion period can be efficiently reduced by increasing the electrode/electrolyte contact area (providing more diffusion channels), reducing particle size (minimizing Li^+^ diffusion distances), and exploring 3D structures (increasing dimensional constants) [34, 35]. In short, effective strategies that can improve the LT diffusion rate of Li^+^ involve the exploration of new materials with reduced ${\Delta }G$, the design of appropriate 3D structures, and the reduction of diffusion distances (*x*) through morphological regulation.

### Lithium Deposition

The severe Li deposition is another key factor concerning the LT performance of LIBs. As the temperature decreases from RT, the potential for the lithiation process becomes close to that of Li deposition [36], and the nucleation barrier of Li deposition is lower than the intercalation barrier; therefore, Li deposition onto graphite surface is preferred rather than insertion into the graphite layer [37]. The formation and proliferation of Li dendrites at LT have been studied by Corey et al. [38], and an equation that could reveal the intricate relationship between short-circuit time ($t_{{{\text{sc}}}}$), temperature (*T*), and dendrite morphology, was proposed:

5 $$\begin{array}{*{20}c} {t_{{{\text{sc}}}} = t_{i} f\left( T \right) + \frac{l}{{\upsilon_{d} f\left( {i,T,{\text{morphology}}} \right)}} + {\text{morphology }}\;f\left( T \right)} \\ \end{array}$$

Here, $t_{i}$ represents the time of the first dendrite appeared, $\upsilon_{d}$ denotes the rate of dendrite growth, $l$ is the distance between anode and cathode, and $i$ signifies the applied current. Temperature directly affected the morphology and formation rate of Li dendrites. As the temperature decreased, $t_{i}$ was shortened and $\upsilon_{d}$ was accelerated, resulting in the formation of sharper and more pronounced dendritic-like Li plating [38]. Simultaneously, as a consequence of side reactions associated with Li deposition, the thickness of the solid electrolyte interface (SEI) increased, leading to the accumulation of "dead" lithium, which hindered the diffusion of Li^+^. These factors contribute to a substantial degradation in LT capacity for LIBs [39]. Thus, it is necessary to inhibit the undesired Li deposition; apparently, the key strategies to avoid Li deposition include the development of new electrode materials with higher working potential and the modification of existing electrode materials with lower intercalation overpotential by surface modifications and interface engineering [40].

## An overview of Low-Temperature Anode Materials

**Fig. 2:**
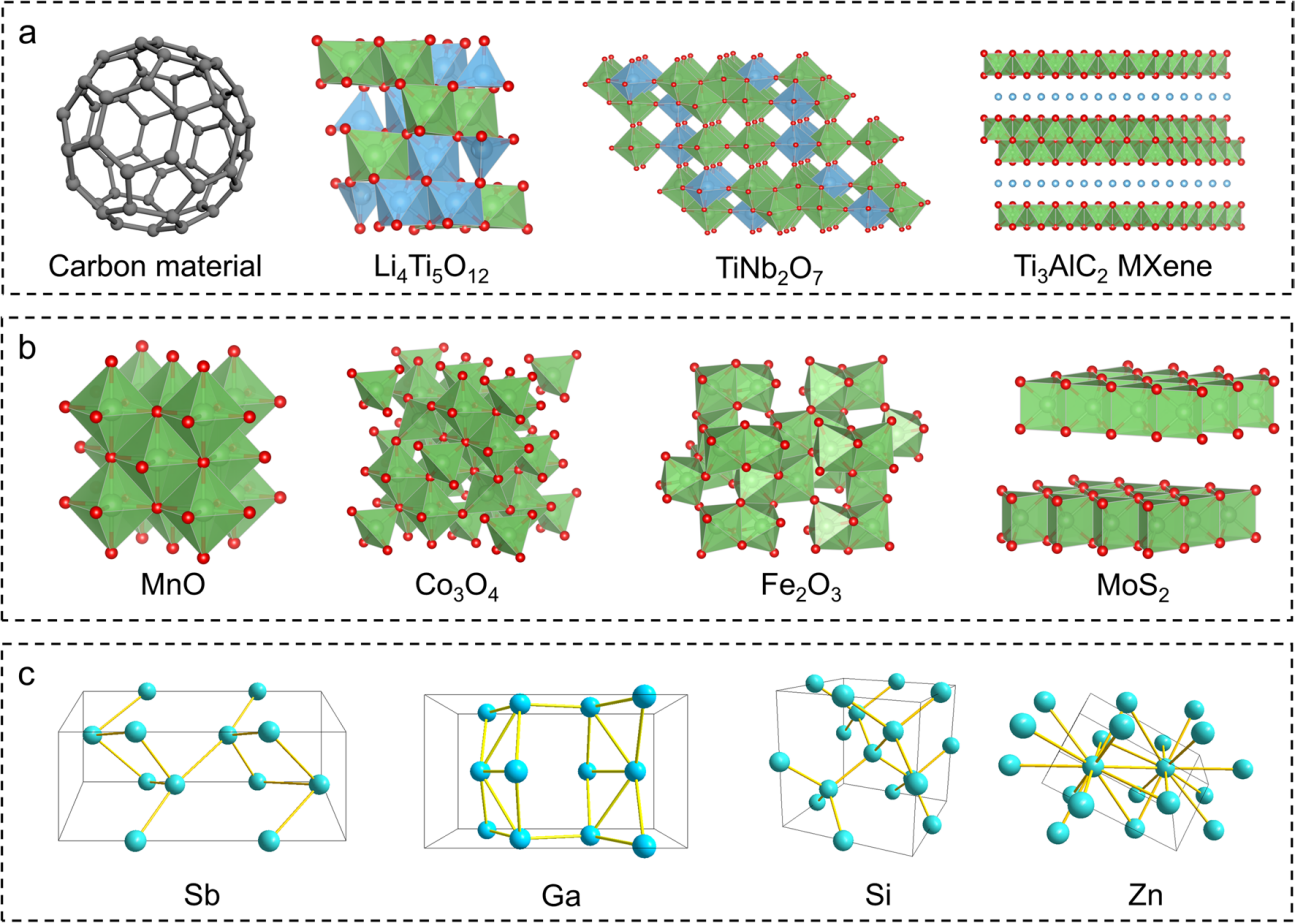
Crystal structure of typical **a** intercalation-typed compounds, **b** conversion-typed compounds, and **c** alloy-typed compounds

Obviously, the anode materials play a crucial role in determining the LT performance of LIBs. Apart from the commercially employed graphite anodes, many other materials toward promising LT electrochemical performance (enhanced gravimetric specific capacity, high operating voltage, and extended life span) have been reported in recent years [10]. These materials can be classified into three fundamental types based on the energy storage mechanisms [41]: intercalation-typed materials, conversion-typed materials, and alloy-typed materials (Fig. 2). Following, a brief introduction concerning their advantages and disadvantages at LT for each category is presented.

### Intercalation-Typed Compounds

Generally, intercalation-based materials exhibit a reversible Li^+^ storage through intercalation and deintercalation processes, which can be described by Eq. 6:

6 $$\begin{array}{*{20}c} {M + y{\text{Li}}^{ + } + y{\text{e}}^{ - } \leftrightarrow {\text{Li}}_{{\text{y}}} M} \\ \end{array}$$

where *M* represents a random intercalation-typed material. Microscopically, Li^+^ can be inserted into and subsequently extracted from these materials without intrinsic structure transformation or notable volumetric change; this characteristic endows the intercalation-typed anodes with reasonable cycling stability and excellent capacity retention under high-rate charge/discharge process.

Carbon-based materials are typical intercalation-typed anode materials for commercial LIBs [42, 43], which offer balanced electrochemical performances at acceptable costs. Besides the widely used graphite material, other carbon materials with different microstructures and morphologies have been explored, such as graphene, hard carbon, carbon nanofibers [44], and carbon nanotubes (CNTs) [18]. For instance, graphene is an attractive 2D-layered carbon-based material consisting of carbon atoms with a hexagonal structure. It exhibits excellent electronic conductivity, high diffusivity, strong mechanical strength, outstanding chemical stability as well as a high theoretical capacity of 744 mAh g^−1^ [45]. Besides, CNTs are 1D carbon-based materials consisting of coiled graphene sheets [46]. The unique topology endows CNTs with excellent properties, such as elevated conductivity, wide surface area, and excellent chemical stability, resulting in improved electrochemical performance. CNTs in single-wall can exhibit an initial discharge capacity of 2390 mAh g^−1^ [47]. However, the capacity of carbon-based materials is severely attenuated at low temperatures, resulting from its low Li intercalation potential (~ 0.1 V vs. Li^+^/Li), severe growth of lithium dendrite, and fast structural deterioration. Thus, it is essential to modify carbon materials or construct carbon-based composite for low-temperature LIBs.

Furthermore, some transition metal oxides (titanium oxides, and niobium oxides, etc.) can act as intercalation-typed materials for lithium storage. For example, anatase TiO_2_ undergoes three steps: solid solution, phase transformation (~ 1.75 V vs. Li^+^/Li), and interfacial storage during the Li intercalation process [48]. Then, Li_4_Ti_5_O_12_ is considered a promising LT anode material, which possesses a theoretical capacity of 175 mAh g^−1^ and a flat operating potential of 1.55 V (vs. Li^+^/Li). The high operation potential of Li_4_Ti_5_O_12_ holds benefits for mitigating the formation of the SEI layer together with the Li^+^ deposition process [49]. Moreover, the volumetric change of Li_4_Ti_5_O_12_ (LTO) during the charge/discharge process is about 0.2%, enabling its superior structural stability and extended lifespan at low temperatures. Li_4_Ti_5_O_12_ nanoparticles can exhibit a discharge capacity of 83 mAh g^−1^ at − 30 °C, surpassing that of commercial graphite [50]. Related niobium oxides, such as Nb_16_W_5_O_55_ [51] and TiNb_2_O_7_ [52], introduce more redox electron pairs during the lithiation and delithiation process, leading to higher specific capacities upon use as anodes. Specifically, TiNb_2_O_7_ (TNO) can exhibit a theoretical capacity of 388 mAh g^−1^ by a four-electron redox reaction. However, the insertion of much lithium ions within the lattice results in a higher volumetric change of 5.5% during the charge/discharge process. When applied these transition metal oxides in LT environment, lithium plating during charging process can be avoided even at a large current density, resulting from its high lithium insertion potential. A reversible discharge capacity of 76.6 mAh g^−1^ (0.2 A g^−1^) is obtained after 200 cycles at − 20 °C for porous TiNb_2_O_7_ microsphere [53]. However, its inherent low electronic conductivity and Li^+^ diffusivity are more significant at low temperature, which seriously limits its further application. Thus, it is essential to modify the crystal structure and reduce particle size to improve its ion and electron transfer rates, leading to enhanced LT kinetics.

Besides, the MXene-based family, which is an emerging kind of 2D transition metal carbides and nitrides [54], can also be used as LT anode materials. Due to the rapid Li^+^ intercalation process caused by their large interlayer spacings, MXene materials deliver favorable electrochemical performance [55]. A modified Ti_3_C_2_T_z_ MXene anode could exhibit an initial capacity of 385 mAh g^−1^ and maintain 213 mAh g^−1^ at − 10 °C [56].

### Conversion-Typed Compounds

Many transition metal oxides possess the ability to store lithium via conversion reactions. Specifically, these metal oxides undergo a transformation into metallic clusters and Li_2_O during the lithiation process:

7 $$\begin{array}{*{20}c} {{\text{M}}_{{\text{x}}} {\text{O}}_{{\text{y}}} + 2y{\text{Li}}^{ + } + 2y{\text{e}}^{ - } \leftrightarrow y{\text{Li}}_{2} O + xM} \\ \end{array}$$

Here, *M* signifies a metal element. Currently, an extensive array of transition-metal oxides [57] (Fe_2_O_3_, MnO, and Co_3_O_4_, etc.) and sulfides [58] (MoS_2_, FeS, and WS_2_, etc.) have been developed as conversion-type anodes. Compared to intercalation-typed anodes, conversion-typed anodes generally exhibit higher specific capacities. For instance, MnO is endowed with a theoretical specific capacity of 755 mAh g^−1^ and a moderate discharge potential of 0.5–0.6 V (vs. Li^+^/Li) [59]; Co_3_O_4_ exhibits a theoretical capacity of 890 mAh g^−1^ and a lithiation potential of 1.1 V (vs. Li^+^/Li) [60]. However, due to continuous phase transitions during the cycling process, the conversion-typed anodes also experience irreversible structural deterioration and large volume expansion (~ 200%), resulting in poor rate performance and notable capacity fading. For instance, MnO@MnFe_2_O_4_ anode delivered a high initial capacity of 1493 mAh g^−1^, yet it severely decreased to 368 mAh g^−1^ only after 10 cycles [61]. When applied these conversion-typed transition metal oxides in LT environment, a significantly excellent capacity can be obtained. For example, a reversible discharge capacity of 456 mAh g^−1^ (0.1 A g^−1^) is obtained after 300 cycles at − 25 °C for MnO@Graphite [59]. By adjusting its morphology, a high discharge capacity of 642 mAh g^−1^ (0.2 A g^−1^) can be obtained after 50 cycles at − 25 °C for peony-like holey Co_3_O_4_ [62]. However, the structural degradation resulting from volume expansion and the concerns about decreasing reaction kinetics derived from low temperature seriously hinder its LT cycling performance. To realize superior LT capability, further artificial rational design and construction of suitable composite systems becomes of great importance and urgency.

It is worth noting that some metal sulfides exhibit multiple charge storage mechanisms. For instance, MoS_2_ is a typical transition-metal sulfide and can deliver a high theoretical capacity of 670 mAh g^−1^. The lithiation process of MoS_2_ encompasses two steps [63]. Firstly, MoS_2_ exhibits an intercalation process where Li^+^ can be embedded into the interlayer space to form Li_x_MoS_2_ at 1.0−1.1 V (vs. Li^+^/Li). Then, MoS_2_ exhibits a conversion process in that Li_x_MoS_2_ is transformed into Li_2_S and metallic Mo clusters at 0.5–0.6 V (vs. Li^+^/Li). However, the poor conductivity of metal sulfides and structural deterioration at low temperatures lead to rapid capacity decay and unsatisfactory rate capacity. When applied at − 20 ℃, pure MoS_2_ delivered an initial capacity of 680 mAh g^−1^, but only remained 264 mAh g^−1^ after 200 cycles at 1 A g^−1^ [63].

### Alloy-Typed Compounds

Generally, alloy-typed anodes refer to a range of compounds that store lithium through the alloying/dealloying mechanism. During the lithiation process, these materials react with lithium to form the corresponding lithium-based alloys [64]. The alloying/dealloying mechanism can be described by Eq. 8:

8 $$\begin{array}{*{20}c} {M + y{\text{Li}}^{ + } + y{\text{e}}^{ - } \leftrightarrow {\text{Li}}_{{\text{y}}} M} \\ \end{array}$$

Here, *M* represents a metal element or an alloy compound. Alloy-typed materials mainly encompass the metallic and semimetallic elements within the IVA and VA groups [65]. These elements can alloy with multiple lithium ions, resulting in suitable lithium deintercalation potential and higher specific capacities than intercalation-typed materials, which make them a critical choice for extreme conditions. For instance, silicon (Si) is a promising alloy-typed compound, which can react with Li^+^ to form Li_4.4_Si. It has many merits, such as high theoretical specific capacities (4200 mAh g^−1^), abundant raw material availability, and environmental friendliness [66]. Si anode could deliver a discharge capacity of 1000, 850, and 440 mAh g^−1^ at 20, 10, and 0 °C, respectively [67]. Similarly, metallic tin (Sn) undergoes lithiation up to the terminal compound of Li_4.4_Sn, leading to a theoretical capacity of 994 mAh g^−1^ [68]. A discharge capacity of 680, 400, and 250 mAh g^−1^ for Sn anode was obtained at  − 10, − 30, and − 40 °C, respectively [69]. It should be noted that alloy-typed compounds always experience huge volumetric fluctuations (about 300%) during the alloying/dealloying process [70], which accelerates the formation of cracks and the disintegration of materials and thus impacts the cycling stability. Due to the slow dynamics at low temperatures, these disadvantages can be amplified, leading to severe capacity attenuation. Consequently, the Sn anode underwent a severe capacity attenuation at − 40 °C, and there is almost no capacity after 25 cycles [69]. Its LT performance can be improved by constructing composite systems and applying nanoscale technology.

In conclusion, the aforementioned three types of anode materials have their advantages and disadvantages and face significant challenges for practical LT application. Firstly, for the intercalation-typed carbon-based materials, the prevailing choice for commercial batteries, their performance will be severely decayed at low temperatures, resulting from the accelerated growth of lithium dendrite. It is feasible to improve its LT performance by engineering surface groups and constructing carbon-based composite. Besides, some emerging intercalation-typed transition metal oxides exhibit higher lithiation potential, which is beneficial for preventing the formation of lithium dendrites, and their small volumetric changes ensure exceptional cycle stability at low temperatures, yet the low theoretical capacity and insufficient intrinsic electronic conductivity limit their large-scale applications in LT LIBs. Thus, current studies on these materials aim at the enhancement of electronic conductivity and the construction of composites, including dimension control, surface modification, and lattice regulation.

For the conversion-typed anodes, they can offer moderate theoretical specific capacities (typically over 500 mAh g^−1^) at low temperatures; however, the continuous phase transition, irreversible structural deterioration, and large volumetric expansion lead to a severe capacity attenuation during the cycles at low temperatures. In this context, studies focusing on the development of composites that integrate metal oxides or sulfides with carbon-based materials are ongoing, which can effectively improve the mechanical integrity and the electronic conductivity, while mitigating the volume expansion for these conversion-typed materials.

For the alloy-typed anodes, they can deliver significantly higher theoretical specific capacity than other kinds of materials, enabling the potential for thinner active material layers. However, the huge volume changes during the alloying/dealloying process seriously impact its cyclic stability. Thus, the key to modifying this alloy-typed compound remains to mitigate the enormous volume change, leading to the exploration of porous materials with large void spaces and the construction of composite materials with supporting skeletons.

## Modification Strategies

**Fig. 3:**
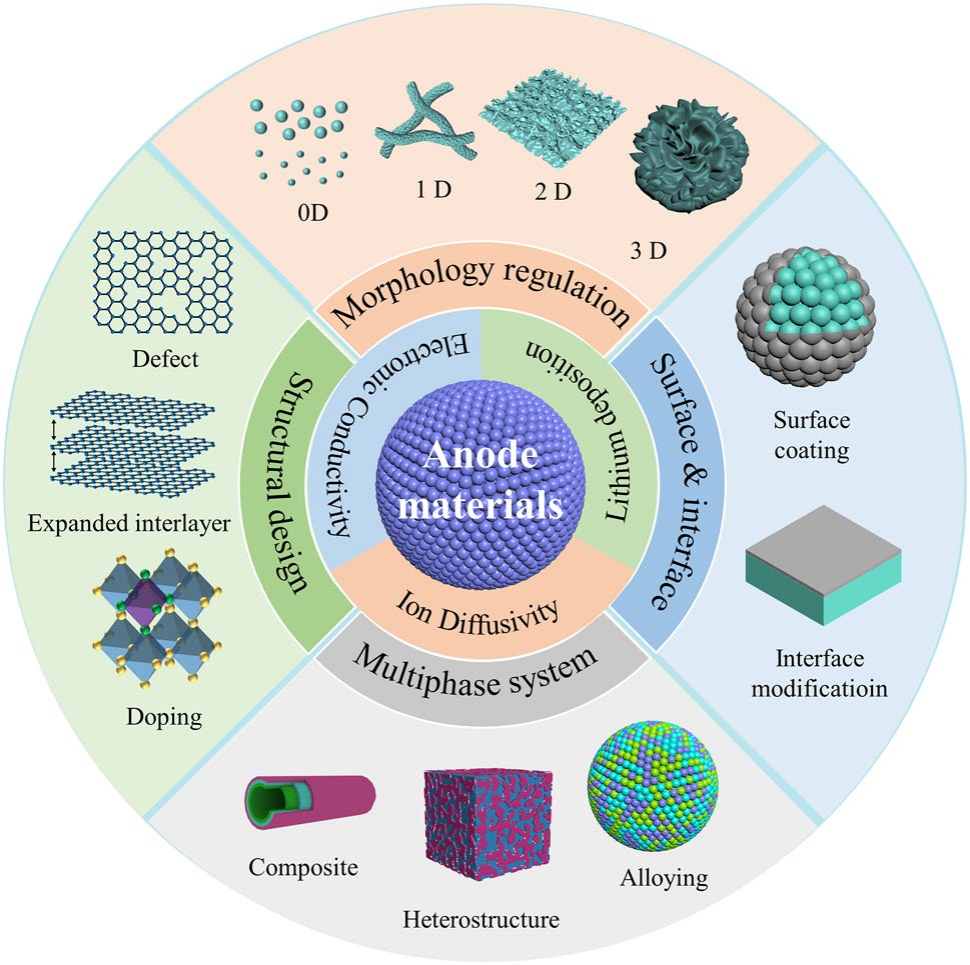
Strategies for microstructural regulations of LT anode materials

Recently, significant achievements have been realized in developing high-performance LT anodes based on intercalation-typed, conversion-typed, and alloy-typed compounds. In the following section, we will try to perform a comprehensive summary of recent progress in LT anode materials, with our highlight, especially on the state-of-the-art modification strategies. These strategies will be classified into four categories, which are morphology regulation, structural design, surface & interface engineering, and multiphase system. An overview of these modification strategies is presented in Fig. 3.

### Morphology Regulation

The particle size and morphology of anode materials play a remarkable role in determining their LT electrochemical performance, which makes the microstructural regulation of these materials a popular strategy [71]. Fundamentally, this approach holds many benefits for improving the kinetics of Li^+^ diffusion by diminishing the Li-ion transportation path, mitigating the electrode polarization, and increasing the surface area of electrode materials [72, 73]. Therefore, anode compounds with diversified morphologies, including zero-dimensional (0D) nanoparticles, 1D nanowires and nanotubes, 2D nanosheets, and 3D complex nanostructures, have been applied in LIBs [74]. The representative anode materials with controlled morphologies together with their LT electrochemical performances are listed in Table 1.

**Table 1:** Summary of anodes with modulated morphologies and their LT performances

Morphology regulation	Anode materials	Potential range/V	Mass loading/mg cm^−2^	Capacity/mAh g^−1^	Current density/A g^−1^	Test temperature/°C	Capacity retention ratio/vs. RT (%)	Electrolyte/volume ratio	References
0D	Carbon nanosphere	0.01–3.0	~ 12.4	160	0.1	− 35	20	1 M LiPF_6_ in EC/DEC = 1:3	[75]
0D	Li_4_Ti_5_O_12_	1.0–3.0	~ 2.1	83	0.13	− 30	51	1 M LiPF_6_ in PC/DME = 1:1	[50]
0D	Li_4_Ti_5_O_12_	1.1 − 2.5	~ 6.0	109	0.17	− 20	64	1 M LiPF_6_ in EC/DMC = 1:1	[76]
0D	Rutile TiO_2_	0.1–3.0	~ 2.0	77	0.07	− 40	24	1 M LiPF_6_ in EC/DMC/DEC = 1:1:1	[77]
0D	Nanoscale Nb_2_O_5_	1.0–3.0	~ 1.0	121	0.01	− 75	64	5 M LiTFSI in EA/DCM = 1:4	[22]
0D	Nano SnO_2_	0.01–3.0	–	423	0.2	− 30	50	1 M LiPF_6_ in EC/PC/EMC = 1:1:2 with 5% FEC	[78]
0D	Ge particles	0.005−1.5	–	566	0.65	− 20	39	1.3 M LiPF_6_ in EC/DEC = 3:7 with 10% FEC	[79]
1D	H_2_Ti_2_O_5_ nanotubes	1.0 − 2.5	~ 3.0	100	0.34	− 25	50	1 M LiPF_6_ in EC/DMC = 1:3	[80]
1D	Li_3.9_Cr_0.3_Ti_4.8_O_12_ nanofibers	1.0 − 2.5	~ 1.5	100	0.17	− 20	61	1 M LiPF_6_ in EC/DEC = 1:1	[81]
1D	Ge nanowires	0.01–3.0	~ 0.06	255	1.5	− 50	22	1 M LiClO_4_ in PC/DME = 7:3	[82]
2D	S/F carbon nanosheet	2.5–4.0	~ 3.0	63	0.1	− 10	46	1 M LiPF_6_ in EC/DEC = 1:1 with 5% FEC	[83]
2D	MoS_2_@graphene	0.01–3.0	–	720	0.1	− 20	66	1 M LiPF_6_ in EC/EMC/DMC = 1:1:1	[84]
2D	PGN@CNT	0.01−1.5	~ 2.0	125	0.04	− 20 ℃	34	1 M LiPF_6_ in EC/EMC/DMC = 1:1:1	[85]
2D	GeO_x_@MXene	0.01−1.5	~ 1.0	334	0.24	− 40	28	1 M LiPF_6_ in EC/DEC = 1:1 with 10% FEC and 2% VC	[86]
3D	graphitic tubular foam	0.005–3.0	–	136	0.04	− 20	16	1 M LiBF_4_ in EC/EMC/DMC = 1:1:1	[87]
3D	Peony-like Co_3_O_4_	0.01–3.0	~ 0.9	1173	0.2	− 25	62	1 M LiPF_6_ in EC/EMC/DMC = 1:1:1	[62]
3D	Coral-like Fe_7_Se_8_@C	0.01–3.0	–	462	1.0	− 25	53	1 M LiPF_6_ in EC/DMC = 1:1	[88]
3D	Hollow Fe_2_(MoO_4_)_3_	0.01–3.0	~ 5.0	281	1.0	− 20	29	1 M LiPF_6_ in EC/DMC = 1:1 with 5% FEC	[89]

#### 0D Nanoparticles

As discussed above, reducing particle size to nanoscale is effective in improving the LT performance of anode materials. Generally, nanoparticles are beneficial to shorten the diffusion path, increase the active area, and provide more lithium storage sites. For example, a carbon nanosphere anchored on the carbon framework was prepared. Its unique positive curvature surface enhanced the Li adsorption on the curved surface and facilitated the Li insertion process at low temperatures. Thus, the carbon nanospheres exhibited a discharge capacity of 160 mAh g^−1^ (0.1 A g^−1^) after 200 cycles at − 35 °C [75]. Allen et al. [50] reported that Li_4_Ti_5_O_12_ (LTO) materials with a diameter of 350 nm manifested an enhanced capacity compared to their 700-nm counterparts. This result could be attributed to the smaller diffusion lengths and larger specific surface area for the 350-nm sample. Phjalainen et al. [76] studied the LT electrochemical performance of LTO particles with various sizes, which were prepared through different grinding methods. The results unveiled that LTO material with smaller particles displayed a discharge capacity of 109 mAh g^−1^ at − 20 °C, while anode with larger particle sizes only delivered 83 mAh g^−1^ under the same conditions. This discrepancy was attributed to the larger surface area, which provided abundant surface reaction sites and shortened diffusion distances, thereby facilitating enhanced LT performance. Moreover, nanosized rutile TiO_2_ [77], Nb_2_O_5_ [22], SnO_2_ [78], and mesoporous Ge particles [79] also showed significantly improvements in the LT performance. Among them, SnO_2_ with a size of about 30 nm demonstrated an enhanced capacity retention of 86.6% (0.2 A g^−1^, after 100 cycles) at − 30 °C, surpassing the samples with larger particle sizes; such an improvement can be attributed to the establishment of faster Li^+^ transfer channels via the inhibition of SnO_2_ grain coarsening [78].

#### 1D Nanomaterials

**Fig. 4:**
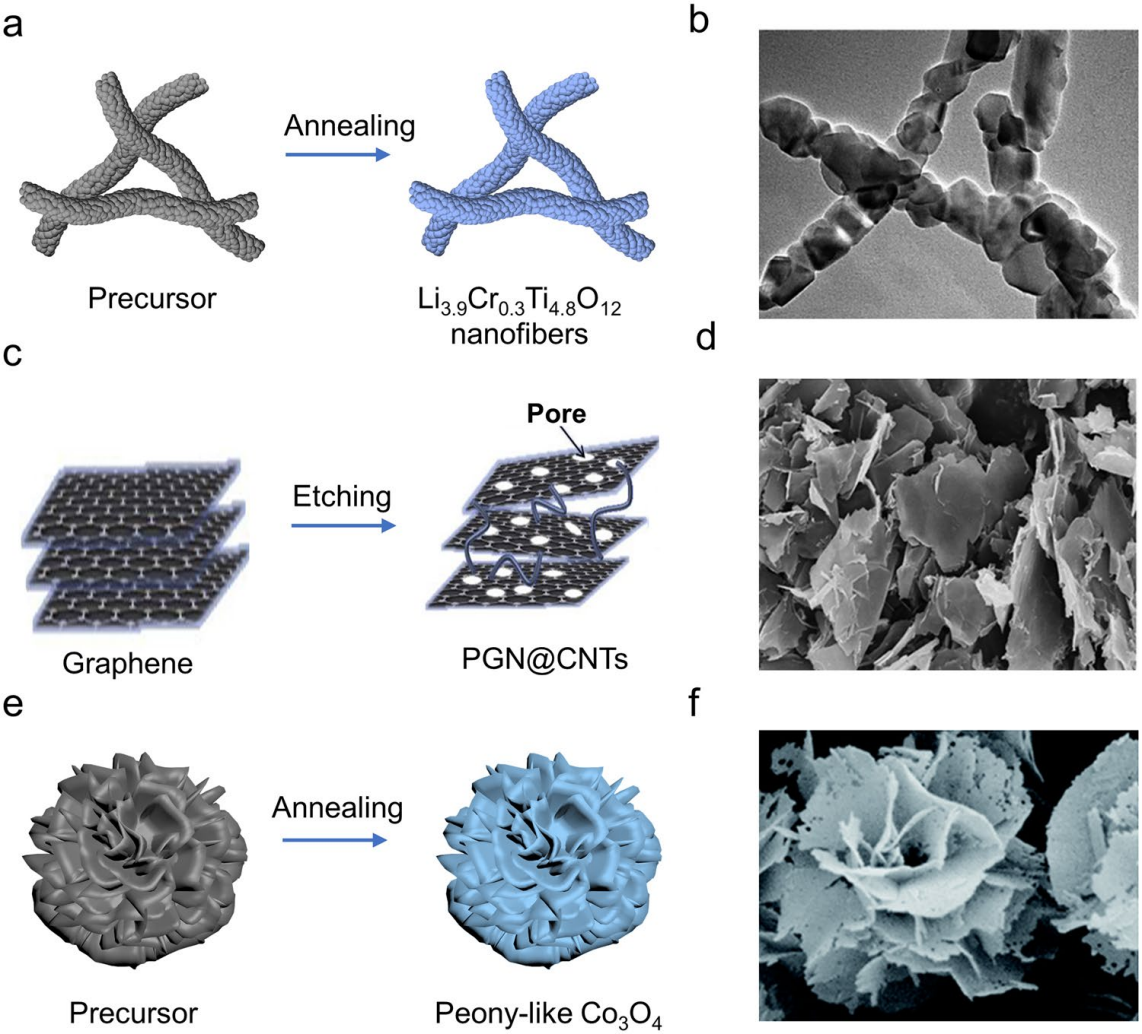
Typical morphology modulated anode materials. **a** Schematic diagram of the synthetic route and **b** scanning electron microscope (SEM) image of Li_3.9_Cr_0.3_Ti_4.8_O_12_ nanofibers. Copyright 2017, Elsevier [81]. **c** Schematic illustration of the preparation process for PGN@CNT and **d** corresponding SEM image. Copyright 2019, Elsevier [85]. **e** Schematic diagram of the synthesis and **f** SEM image of peony-like Co_3_O_4_. Copyright 2019, Royal Society of Chemistry [62]

1D nanomaterials, such as nanofibers and nanowires, have gained significant attention in the electrochemistry field owing to their special structure–performance relationship. For instance, Li et al. [80] synthesized hydrogen titanate (H_2_Ti_2_O_5_) nanotubes via a hydrothermal reaction, featuring a diameter of approximately 9 nm and a length of several hundred nanometers. The nanotubular morphology endows this material with a large specific surface area and facilitates the diffusion of Li^+^ along the nanotube. It leads to a high ion diffusion coefficient even at low temperatures, resulting in a high capacity of 100 mAh g^−1^ at − 25 °C. Then, electrospinning is commonly employed for the fabrication of 1D nanofibers [90, 91]. For example, Zou et al. [81] prepared Li_3.9_Cr_0.3_Ti_4.8_O_12_ nanofibers by introducing some Cr elements (Fig. 4a, b). 1D nanostructure played a positive role in facilitating the diffusion of Li^+^ along the radial direction, thereby substantially reducing the diffusion distance of Li^+^. Consequently, the nanofibers attained a capacity of 100 mAh g^−1^ (0.17 A g^−1^) at − 20 °C. Then, Gavrilin et al. [82] fabricated germanium nanowires (Ge NWs) on titanium foils through an electrodeposition process, with a length of ~ 500 nm and a diameter of 20–40 nm. Consequently, the 1D feature of Ge NWs facilitated the ion transportation process, leading to an excellent RT capacity (1.5 A g^−1^, 1300 mAh g^−1^) as well as a favorable LT capacity (− 50 °C, 255 mAh g^−1^).

#### 2D Nanomaterials

To effectively increase the constant of dimensionality and shorten the diffusion time, 2D nanomaterials have been proposed. For instance, S/F co-doped carbon nanosheets exhibited a discharge capacity of 62.6 mAh g^−1^ (0.1 A g^−1^) at − 10 °C [83]. Teng et al. [84] developed a MoS_2_/graphene nanostructure (MoS_2_/G) using a hydrothermal method, where MoS_2_ nanosheets were vertically grown along graphene nanosheets. This MoS_2_/G material exhibited a substantial increase in surface area, thereby improving the reaction sites and reducing the diffusion distance of Li^+^. Consequently, a capacity of 720 mAh g^−1^ (− 20 °C, 100 mA g^−1^) was achieved, representing 70% of its RT capacity. As depicted in Fig. 4c, d, Xu et al. [85] developed an advanced carbon-based composite (PGN@CNT), which was composed of carbon nanotubes and thin porous graphite nanosheets. The microstructural characteristics of PGN@CNT shortened the diffusion path and facilitated the Li^+^ transport kinetics, leading to a superior LT performance (125 mAh g^−1^ at 0.04 A g^−1^, − 20 °C). Recently, the emergence of 2D MXenes has sparked considerable interest in their potential applications within the field of LIBs. The key driving factor behind this interest lies in their expanded surface area and high electronic conductivity. A two-dimensional GeO_x_@MXene composite, comprising 2D Ti_3_C_2_ MXene and an ultrathin layer of amorphous germanium oxide (GeO_x_), was synthesized by a wet chemical process [86]. The GeO_x_@MXene material exhibited exceptional electronic conductivity and rapid ion transport, due to its 2D layered structures, demonstrating an outstanding low-temperature capacity of 334 mAh g^−1^ at 0.24 A g^−1^ under − 40 °C.

#### 3D Nanomaterials

Nanomaterials with 3D architecture can offer obvious advantages in terms of high-dimensional constant (*q* = 6), effectively shortening Li^+^ diffusion distance, preventing the agglomeration of active particles, and enlarging electrode–electrolyte effective contact. Firstly, a branched N-doped graphitic tubular foam was prepared by retaining the structural outlines of the 3D SiO_2_ template [87]. A reversible capacity of 135.8 mAh g^−1^ was obtained at − 20 °C, resulting from its 3D tubular framework and larger layer spacing of graphite. As displayed in Fig. 4e, f, a typical example of 3D material is the peony-like holey Co_3_O_4_ reported by Duan et al. [62]. The 3D ultrathin nanostructures with vertically perforated paths favored the reduction of Li^+^ diffusion pathways and the improvement of reaction kinetics, leading to an impressive initial LT capacity of 1173 mAh g^−1^ at 0.2 A g^−1^ and a reversible capacity of 642 mAh g^−1^ after 50 cycles at − 25 °C. Then, Fan et al. [88] prepared a coral-like Fe_7_Se_8_@C anodes that exhibited exceptional performance under LT conditions. At − 25 °C, it delivered a capacity of 461.8 mAh g^−1^ (1 A g^−1^). The 3D morphology regulation of Fe_7_Se_8_@C endowed the composite with plentiful ion/electron-transport routes. Consequently, the storage kinetics and diffusion kinetics of Li^+^ at low temperatures were significantly improved. Fe_2_(MoO_4_)_3_ with a hollow microstructure was successfully synthesized by a bubble-templated method [89]. The unique hollow multistage microspheres offered several advantages for enhanced electrochemical activity in low-temperature environments: (1) The reduced diffusion length facilitated ion and electron transport, leading to accelerated reaction kinetics; (2) the hollow architecture effectively mitigated volume changes and suppressed structural degradation, resulting in outstanding LT performance (281 mAh g^−1^ at 1 A g^−1^, − 20 °C).

### Structural Design

To improve the LT performance of anode materials, it is also an effective strategy to employ structural design. This approach entails the deliberate modification of the crystal structure to optimize its intrinsic electronic conductivity and Li^+^ diffusivity. Key methods encompass expanding interlayer spacing, doping with heteroatoms, and engineering lattice defects. Typical anode materials with excellent LT performances via microstructural engineering are listed in Table 2.

**Table 2:** Summary of typical anodes with different structural design strategies and their low-temperature performance

Structural design	Anode materials	Potential range/V	Mass loading/mg cm^−2^	Capacity/mAh g^−1^	Current density/A g^−1^	Test temperature/°C	Capacity retention ratio/vs. RT	Electrolyte/volume ratio	References
Expanding interlayer spacing	Mesocarbon microbead	0.01−1.5	~ 3.0	100	0.08	− 40	–	–	[92]
Expanding interlayer spacing	MoS_2_/carbon	0.01–3.0	~ 1.5	854	0.1	− 20	73%	1 M LiPF_6_ in EC/DMC = 1:1	[63]
Expanding interlayer spacing	Ni_2_Nb_34_O_87_	0.8–3.0	~ 1.1	207	0.04	− 10	61%	1 M LiPF_6_ in EC/DMC/DEC = 1:1:1	[93]
Doping	Nb-doped Li_4_Ti_5_O_12_/TiO_2_	1.0 − 2.5	~ 1.5	128	0.34	− 20	86%	1 M LiPF_6_ in EC/EMC/DMC = 1:1:1	[94]
Doping	Co-doped Zn_2_SnO_4_/graphene	0.01–3.0	~ 1.0	196	0.12	− 25	23%	1 M LiPF_6_ in EC/EMC/DMC = 1:1:1	[95]
Doping	W-doped Li_4_Ti_5_O_12_/TiO_2_	1.0–3.0	~ 1.5	195	0.1	− 20	81%	1 M LiPF_6_ in EC/EMC/DMC = 1:1:1	[96]
Doping	N-doped TiO_2_/TiN/graphene	1.0–3.0	~ 1.5	211	0.1	− 20	65%	1 M LiPF_6_ in EC/DMC = 1:1	[97]
Doping	La- and F-doped Li_4_Ti_5_O_12_	0.5 − 2.5	–	100	0.17	− 20	57%	1 M LiPF_6_ in EC/DEC = 1:1	[98]
Defects	Partially reduced TiNb_24_O_62_	1.0–3.0	~ 1.0	313	0.04	− 20	83%	1 M LiPF_6_ in EC/DMC/DEC = 1:1:1	[99]
Defects	Dual-phase TiO_2_	0.01–3.0	~ 1.0	120	0.34	− 25	48%	1 M LiPF_6_ in EC/DEC = 1:1 with 5% FEC	[100]
Defects	Crumpled graphene	0.01 − 2.8	–	48	0.01	− 60	13%	1 M LiPF_6_ in EC/EMC/MP = 2:6:2	[101]

#### Expanding the Interlayer Spacing

Expansion of interlayer spacings for layered materials is regarded as an effective strategy to address their inherent limitations associated with the slow kinetics for Li^+^ diffusion. By doing this, the ion diffusion barrier of Li^+^ can be reduced, thereby promoting the Li^+^ insertion kinetics and ultimately enhancing the electrochemical performance at low temperatures [102]. Zhao et al. [92] proposed a mesocarbon microbead material (MCMB) with expanded layer spacing, which delivered a remarkable capacity of 100 mAh g^−1^ at − 40 °C, surpassing that of commercial mesocarbon microbeads in identical conditions.

**Fig. 5:**
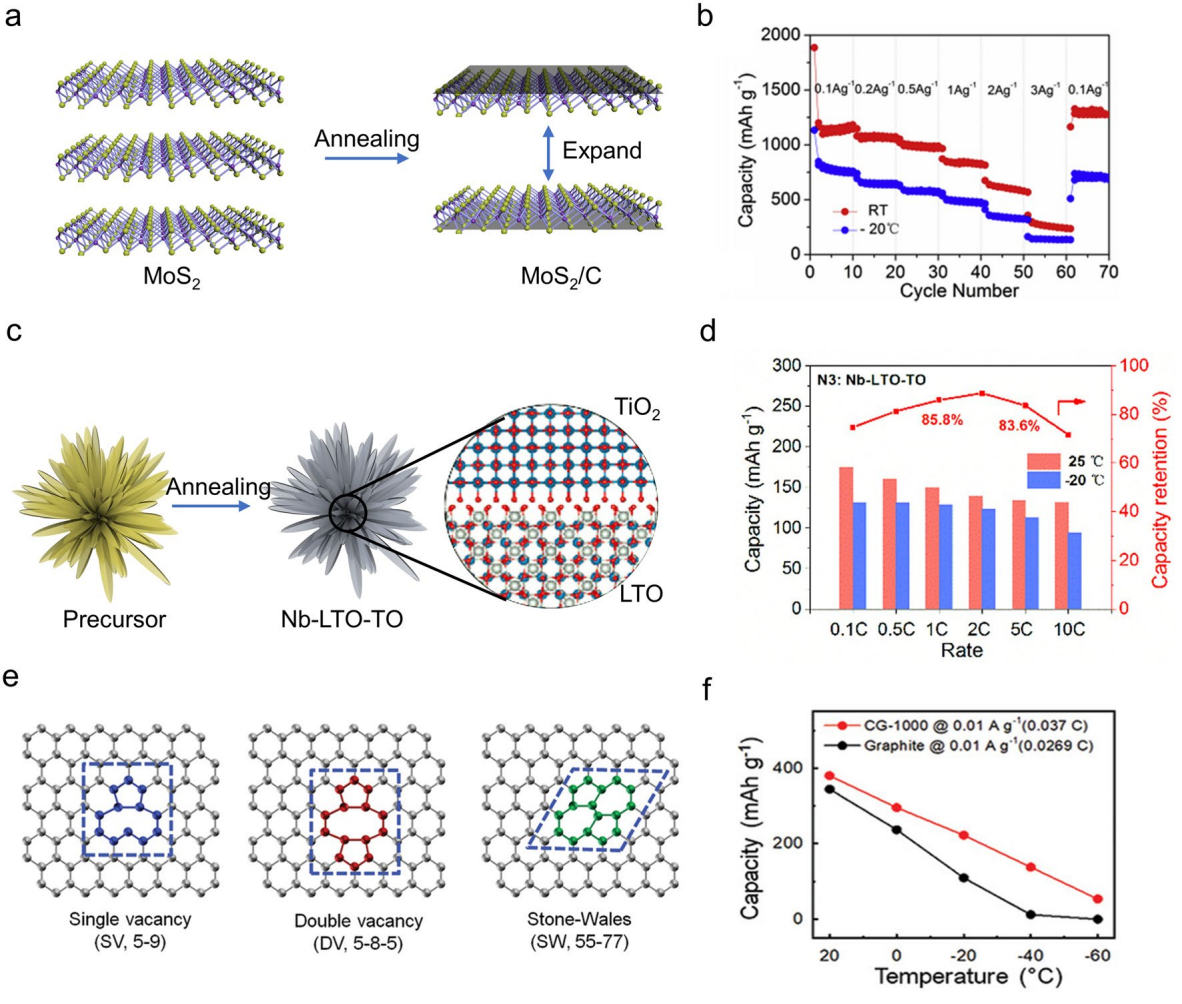
Examples of structural design on anode materials for LT LIBs. **a** Schematic illustration of MoS_2_/C and **b** its rate performance. Copyright 2020, Elsevier [63]. **c** Preparation process of NLTO and **d** its corresponding electrochemical performance. Copyright 2021, Elsevier [94]. **e** Schematic illustration of crumpled graphene with different defects: single vacancy (SV), double vacancy (DV), and Stone–Wales (SW). **f** LT performance of graphite and crumpled graphene at 0.01 A g^–1^. Copyright 2021, Wiley–VCH [101]

Moreover, the LT performance of metal sulfide/oxide anodes could also be significantly improved by expanding the lattice constant. An example refers to the synthesis of a MoS_2_/C composite with increased layer spacing through a soft-template method [63] (Fig. 5a, b). Consequently, this modified MoS_2_ facilitated the diffusion of Li^+^, and the incorporation of a surface carbon layer improved the electronic conductivity. Thus, it demonstrated a specific capacity of 854 mAh g^−1^ (0.1 A g^−1^) and 140 mAh g^−1^ (3 A g^−1^) at − 20 °C, while maintaining a capacity retention of 95.6% after 50 cycles at 0.1 A g^−1^. Lv et al. [93] designed and prepared a nickel niobium oxide (Ni_2_Nb_34_O_87_, 1 C = 0.39 A g^−1^). Owing to the large lattice constant and abundant free electrons in Ni^2+^, Ni_2_Nb_34_O_87_ exhibited fast Li^+^ diffusion kinetics and high electronic conductivity. Thus, it displayed an initial capacity of 207 mAh g^−1^ (− 10 °C, 0.1 C) and a capacity retention of 64.0% after 1000 cycles (2 C, − 10 °C).

#### Doping

Introducing heteroatoms into the lattices of anode materials presents a feasible approach to tune lattice parameters or lattice defects, which can successively lead to improved intrinsic electronic conductivity, enhanced Li^+^ diffusivity, and finally, enhanced LT electrochemical performance [103, 104]. Transition metal ions with relatively large ionic radii are commonly favored for the above purpose. For example, Meng et al. [94] developed a microspherical Nb-doped Li_4_Ti_5_O_12_/TiO_2_ (NLTO) composite (Fig. 5c, d). The doping of Nb^5+^ caused a partial reduction of Ti^4+^ into Ti^3+^, resulting in expanded lattice parameters and improved conductivity for this composite. Under − 20 °C, the NLTO anode maintained a capacity of 128.6 mAh g^−1^ at 0.34 A g^−1^ and 119.4 mAh g^−1^ at 1.7 A g^−1^. Besides, doping other metal ions into the crystal structure, such as La^3+^ [98], Co^3+^ [95], Mg^2+^ [105], and W^6+^ [96], has also been attempted to enhance their intrinsic electronic conductivity and ion diffusivity.

Apart from metal ions, the incorporation of nonmetal atoms has also shown advantages for enhancing the LT performance of anode materials. Li et al. [97] developed an N-doped TiO_2_@TiN@graphene nanocomposite (NTT/G). The incorporation of N atoms into the TiO_2_ lattice has enhanced the electronic conductivity of NTT/G, leading to a high reversible specific capacity (211 mAh g^−1^, 0.1 A g^−1^) and good cycling stability (about 93% within 500 cycles) at − 20 °C. Of course, a co-doping of metal cations and nonmetal anions has also been investigated [98]. Representatively, the introduction of La^3+^ into the LTO structure induced lattice deformation, thereby enhancing the Li^+^ storage capacity; however, this modification exhibited relatively poor stability over prolonged cycling. When F ions were co-doped with La^3+^ into LTO, a stable SEI layer was formed on the material during cycling. The co-doped LTO exhibited an even higher Li^+^ diffusion coefficient, smaller *R*_ct_, and a more stable discharge behavior at − 20 °C.

#### Defects

Lattice defects (vacancies, dislocations, and grain boundaries) are long known to play crucial roles in controlling ion diffusion behaviors, which consequently affects the intrinsic conductivity for both electrons and ions in the material [106]. In this context, the introduction of oxygen vacancies via calcination under a reducing atmosphere has been proven to be particularly effective for improving the LT performance of anode materials. Jiang et al. [99] developed a partially reduced TiNb_24_O_62_ (PR-TNO) fibrous material and applied it as a LT anode. Benefiting from the abundant oxygen vacancies caused by the partial reduction, the PR-TNO anode exhibited fast electron and ion transportation kinetics and high electrochemical performance. Specifically, PR-TNO exhibited a capacity of 313 mAh g^−1^ (0.04 A g^−1^) and showed remarkable capacity retention (preserved 99.2% of its initial capacity after 1680 cycles at 2 A g^−1^) at − 20 °C. Moreover, a facile hydrothermal method was employed to produce abundant grain boundaries and oxygen vacancies in titanium oxide with a mixture of TiO_2_(B) and anatase phases [100]. The dual-phased TiO_2_ showed excellent Li^+^ storage and transportation kinetics, resulting in an enhanced LT capacity (0.34 A g^−1^, 120 mAh g^−1^) at − 25 °C. As shown in Fig. 5e, f, Lee et al. [101] prepared a crumpled graphene (CG) with controlled chemical and defect structures. At − 60 °C, it delivered a capacity of 48 mAh g^−1^ (at 0.01 A g^–1^). Density functional theory (DFT) calculations revealed that the graphene with considerable defect fraction could promote the adsorption of Li-ions, thereby encouraging the utilization of the surface-controlled charge storage mechanism. Consequently, the storage kinetics and diffusion kinetics of Li^+^ at low temperatures were significantly improved.

### Surface and Interface Engineering

The purpose of surface & interface engineering is to modify the interface between electrode and electrolyte and facilitate rapid electron/ion transfer. Key approaches include surface coating and interface modification. Both strategies aim to improve the electronic conductivity, relieve the volume change during cycling, accelerate the desolvation process, suppress the interface side reactions, and avoid the growth of lithium dendrite for the LT anode [27]. The typical anode materials with modified surface or interface and their LT performance are listed in Table 3.

**Table 3:** Summary of typical anode materials with modified surface or interface and their LT performance

Modification strategies	Anode materials	Potential range/V	Mass loading/mg cm^−2^	Capacity/mAh g^−1^	Current density/A g^−1^	Test temperature/°C	Capacity retention ratio/vs. RT (%)	Electrolyte/volume ratio	References
Surface coating	Carbon-coated natural graphite	0.005 − 2.5	~ 5	106	0.5	− 10	30	1 M LiPF_6_ in EC/DEC = 3:7	[107]
Surface coating	Carbon-coated Li_4_Ti_5_O_12_	1.0 − 2.5	–	150	0.17	− 20	71	1 M LiPF_6_ in EC/DEC = 1:1	[108]
Surface coating	Carbon-coated modified graphite	0.001−1.5	~ 3	310	0.04	0	86	1 M LiPF_6_ in EC/EMC = 1:1	[109]
Surface coating	Surface-fluorinated Li_4_Ti_5_O_12_	1.0 − 2.5	~ 1.1	100	0.17	− 20	60	1 M LiPF_6_ in EC/EMC = 3:7	[110]
Surface coating	Surface-fluorinated Li_4_Ti_5_O_12_	1.0–3.0	–	65	0.17	− 20	42	1.2 M LiPF_6_ in EC/EMC = 3:7	[111]
Surface coating	Sn-coated graphite	0.01−1.5	~ 2.5	152	0.08	− 30	41	1 M LiPF_6_ in EC/DEC/DMC = 1:1:1	[112]
Interface modification	TiO_2_(B)/ graphite	1.0–3.0	~ 1	106	2	− 30	53	1 M LiBF_4_ in EC/PC/EMC = 1:2:7 with 1% FEC	[113]
Interface modification	SnO_2_/LiF/graphite	0.01–3.0	~ 1	637	0.1	− 50	67	1 M LiPF_6_ in EC/PC/EMC = 1:1:8 with 5% FEC	[114]
Interface modification	Sulfide-rich SiO/C	3.0–4.2	~ 2.6	135	0.02	− 20	73	1 M LiPF_6_ in EC/DMC = 1:1	[115]
Interface modification	Ti_3_C_2_T_x_ MXene	0.01–3.0	–	213	0.2	− 10	47	1 M LiBF_4_ in EC/DE/DMC = 1:1:1	[56]
Interface modification	Ti_3_C_2_T_x_ MXene	0.01–3.0	~ 2	226	0.1	− 20	56	1 M LiPF_6_ in EC/DEC = 1:1	[116]

#### Surface Coating

The application of a conductive coating layer on the particle surface of anode materials is a well-established strategy to enhance their electrochemical performance. Materials with high electronic conductivity, favorable compatibility with anode material, and good chemical stability are selected as the coating layer. Consequently, carbon-based materials are the most common candidates for their cheap price and environmental friendliness [117]. The coating layer can be achieved through various physicochemical methods. For instance, Gunawardhana et al. [107] utilized a chemical vapor deposition process to prepare a carbon-coated natural graphite. This technique ensured a uniform and conformal coating on the surface of natural graphite. It was shown that the carbon coating layer efficiently reduced the growth of Li dendrites by suppressing Li deposition, enhanced the formation of LiC_6_, and formed an optimized SEI layer below − 10 °C, which leads to improved stability for the electrode–electrolyte interface. Li et al. [108] synthesized a carbon-coated Li_4_Ti_5_O_12_ hierarchical porous structure (CP-LTO), while the waste phoenix tree leaves were used as the carbon source. This material with 3 wt% carbon maintained a capacity of 150 mAh g^−1^ at − 20 °C. Cai et al. [109] investigated the modified graphite with a 6.5-nm carbon coating layer (G@TC). This anode showed a considerable improvement in the Li^+^ diffusion rate and provided abundant active sites, resulting in a capacity of 310 mAh g^−1^ at 0 °C. Therefore, the carbon coating technique is proven as an effective and promising approach to improving the LT performance and safety of anode materials in LIBs.

Similarly, it can be useful to coat a conductive metal layer on the surface of anode materials. Nobili et al. [112] developed a Sn-coated graphite (SG) material by a physical vapor deposition. The application of a surface Sn coating layer induced multiple benefits, including improved electronic conductivity and enhanced charge transfer, which led to an enhanced LT performance. Specifically, the SG anode exhibited a capacity of 152 mAh g^−1^ (0.08 A g^−1^) at -30 °C. Alex et al. [118] employed a different anode by coating an Al_2_O_3_ layer on the surface of graphite. The results revealed that the incorporation of the Al_2_O_3_ coating layer contributed to the safety and LT performance by effectively preventing the growth of Li plating.

**Fig. 6:**
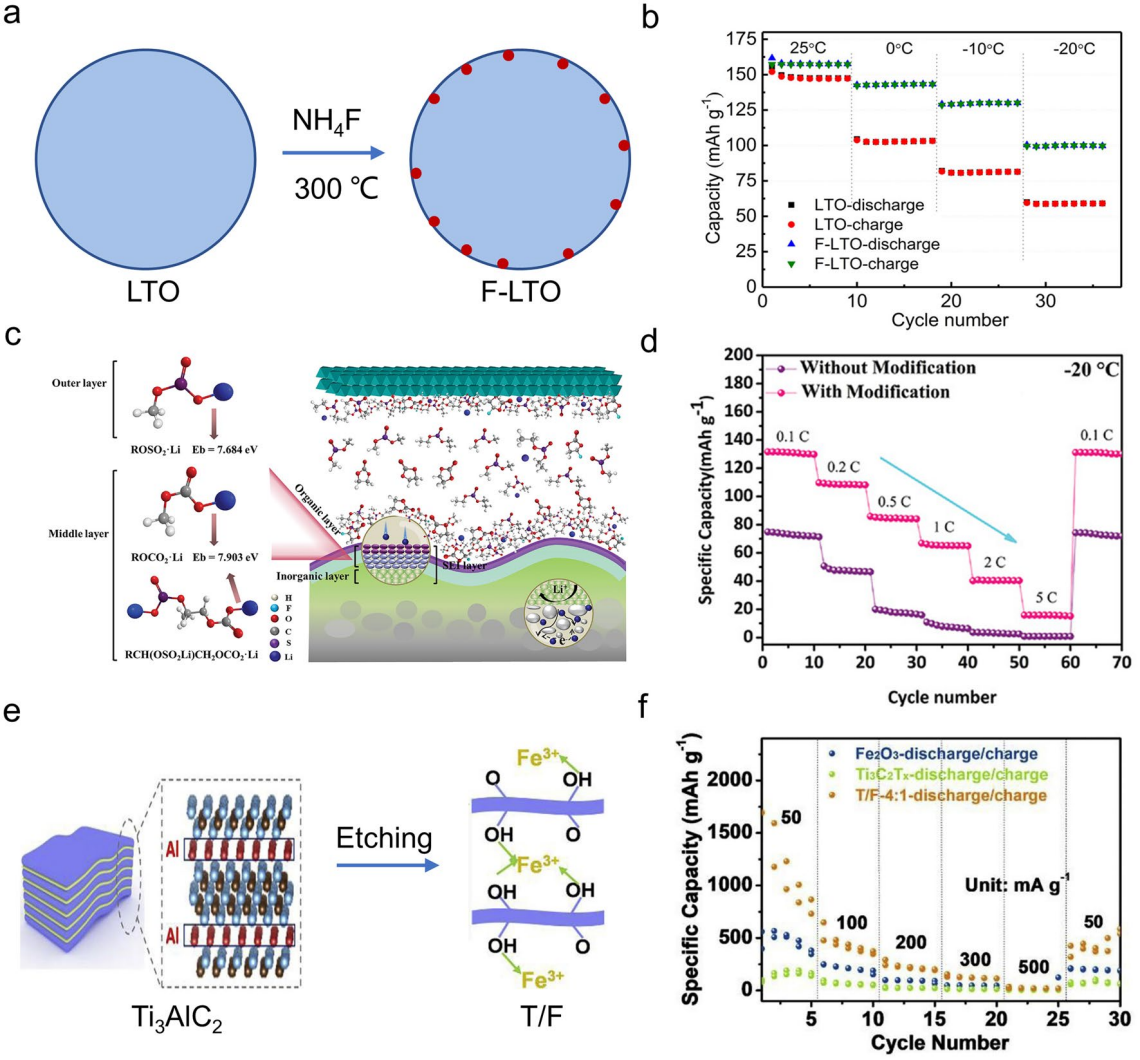
Examples of surface and interface engineering on anode materials for LT LIBs. **a** Schematic for the synthetic process of F-LTO and **b** its capacity at different temperatures. Copyright 2017, American Chemical Society [110]. **c** Binding energy for Li^+^ in the modified layer by DFT and **d** its rate performance at − 20 ℃. Copyright 2022, the Authors [115]. **e** Preparation process of T/F material and **f** its LT rate performance. Copyright 2021, Elsevier [56]

Recently, a surface fluorination technique was also employed to control the surface property of anode materials. Zhang et al. [110] proposed a surface-fluorinated Li_4_Ti_5_O_12_ (F-LTO) material by co-calcination Li_4_Ti_5_O_12_ with NH_4_F (Fig. 6a). LiF was formed on the LTO surface after treatment, which enhanced electronic conductivity, accelerated Li^+^ diffusion, and improved interface stability. The F-LTO anode exhibited a capacity of 100 mAh g^−1^ at − 20 °C, while pristine LTO only delivered 59 mAh g^−1^ at the same conditions (Fig. 6b). Fluorinated material could also be prepared via calcination of the anode material under a fluorine atmosphere. Wang et al. [111] prepared another kind of surface-fluorinated Li_4_Ti_5_O_12_ (FLTO) material by using NF_3_ gas as the calcination atmosphere. The FLTO anode exhibited reduced polarization, enhanced Li^+^ diffusion rate, and improved interfacial stability at low temperatures, and it demonstrated a capacity of 65 mAh g^−1^ after 100 cycles (0.17 A g^−1^) at − 20 °C.

#### Interface Modification

Compared with surface coating, interface modification primarily focuses on optimizing the structure and composition of the SEI layer. For example, Zhang et al. [113] designed a TiO_2_(B)/graphene anode. The incorporation of graphene within TiO_2_(B) could effectively modify its interface, resulting in a reduced R_ct_ and ultimately leading to a reasonable capacity of 106 mAh g^−1^ at − 30 °C. Besides, the addition of LiF in a SnO_2_/graphite composite (SLG) led to the formation of a LiF-rich SEI layer on the electrode/electrolyte interface [114]. The presence of this LiF-rich SEI layer, combined with graphite, effectively mitigated the volume expansion of SnO_2_ nanoparticles during cycling. Furthermore, the formation of the LiF-rich SEI layer also played a crucial role in preserving the stability of the electrode/electrolyte interface, thus contributing to an enhanced LT performance (637.2 mAh g^−1^, 100 mA g^−1^) at − 50 °C. Besides, a sulfide-rich SEI layer was fabricated on the surface of the SiO/C anode [115]. As depicted in Fig. 6c, d, the DFT and molecular dynamics simulation results revealed that the sulfide-rich surface layer and traditional intermediate layer promoted the Li^+^ desolvation process at LT as well as the inner LiF-rich layer accelerated the Li^+^ diffusivity and inhibited dendrite growth. Thus, it exhibited a discharge capacity of 135 mAh g^−1^ (0.02 A g^−1^) at − 20 °C.

The interface properties also could be impacted by the surface functional groups on anode materials. As depicted in Fig. 6e, f, Zhao et al. [56] implanted -O/-OH groups on the surface of MXene material by introducing Fe ions into the Ti_3_C_2_T_x_ suspension and prepared a T/F material, which exhibited a lower diffusion barrier and more active sites for Li^+^. It delivered a reversible capacity of 213 mAh g^−1^ at − 10 °C. Similarly, Wang et al. [116] regulated the terminal surface -O group of Ti_3_C_2_T_x_ MXene (T/O) by calcining it under different atmospheres. The as-prepared partial oxidized T/O anode material exhibited a capacity of 226 mAh g^−1^ at 10 cycles and maintained a capacity of 194 mAh g^−1^ after 1000 cycles at − 20 °C. It demonstrated that the presence of an O-rich surface effectively reduced the Li^+^ migration barrier, enhanced electrolyte wettability, promoted the desolvation process of Li^+^, and facilitated the Li insertion kinetics. These results consistently confirm that the interface modification strategy is beneficial for the LT performance of anode materials.

### Multiphase System

It is now accepted that the construction of a multiphase system as an anode can make use of their respective advantages and achieve an enhanced LT performance. Due to the synergistic effect derived from the components, multi-phased systems may exhibit reduced charge transfer barrier, high electrons/ions conductivity, and sufficient capability to buffer the volume change during cycling [13]. Generally, multiphase systems can be classified into three types: heterostructures, composites, and alloying. The representative multiphase materials and their LT performance as anodes are listed in Table 4.

**Table 4:** Summary of the typical multiphase materials and their LT performance

Modification strategies	Anode materials	Potential range/V	Mass loading/mg cm^−2^	Capacity/mAh g^−1^	Current density/A g^−1^	Test temperature/°C	Capacity retention ratio/vs. RT (%)	Electrolyte/volume ratio	References
Heterostructure	Li_4_Ti_5_O_12_ and rutile TiO_2_	1.0 − 2.5	–	115	0.09	− 40	69	1 M LiPF_6_ in EC/DMC/EMC = 1:1:1	[119]
Heterostructure	Sn@expanded graphite	0.01−2.0	~ 3.0	200	0.06	− 20	31	1 M LiPF_6_ in EC/DMC = 1:1	[120]
Composite	MnO@Graphite	0.01–3.0	–	456	0.1	− 25	40	1 M LiPF_6_ in EC/DEC/DMC/EMC = 1:1:2:1	[59]
Composite	(Nb_2_O_5_/TiNb_2_O_7_)@C	1.0–3.0	~ 3.5	110	0.03	− 40	41	1 M LiPF_6_ in EC/EMC = 1:4	[121]
Composite	Li_4_Ti_5_O_12_@CNTs	1.0−2.3	~ 2.0	140	0.04	− 60	85	0.75 M LiTFSI in DIOX	[122]
Alloying	Cu_18_Zn_82_ alloys	0.005−2.5	~ 3.0	137	0.1	− 30	51	1 M LiPF_6_ in EC/DMC = 1:1	[123]
Alloying	Cu-Ge-Al ternary alloys	0.01–3.0	~ 2.0	122.9	1.0	− 20	47	1 M LiPF_6_ in EC/DMC = 1:1	[124]

#### Heterostructure

The concept of heterostructure herein represents a class of new materials that are integrations of heterogeneous zones with diverse phase structures or properties [125]. Huang et al. [119] prepared a dual-phase heterostructure material (LTO-RTO), wherein Li_4_Ti_5_O_12_ and rutile TiO_2_ were combined. This heterostructure exhibited a reduced activation energy and mitigated volumetric change during cycling. Consequently, it delivered a capacity of 115 mAh g^−1^ at − 40 °C, which was distinctly superior to the pristine LTO electrode. Another heterostructure was prepared via a carbonaceous matrix strategy by Yan et al. [120], wherein Sn nanoparticles were uniformly embedded into the interlayer spacings of expanded graphite (Sn/EG), resulting in the formation of a stacked structure. Similarly, the Sn/EG anode exhibited lower overpotential, higher electronic conductivity, and shorter diffusion distance of Li^+^. It demonstrated an improved capacity of 200 mAh g^−1^ (0.06 A g^−1^) at − 20 °C.

#### Composite

**Fig. 7:**
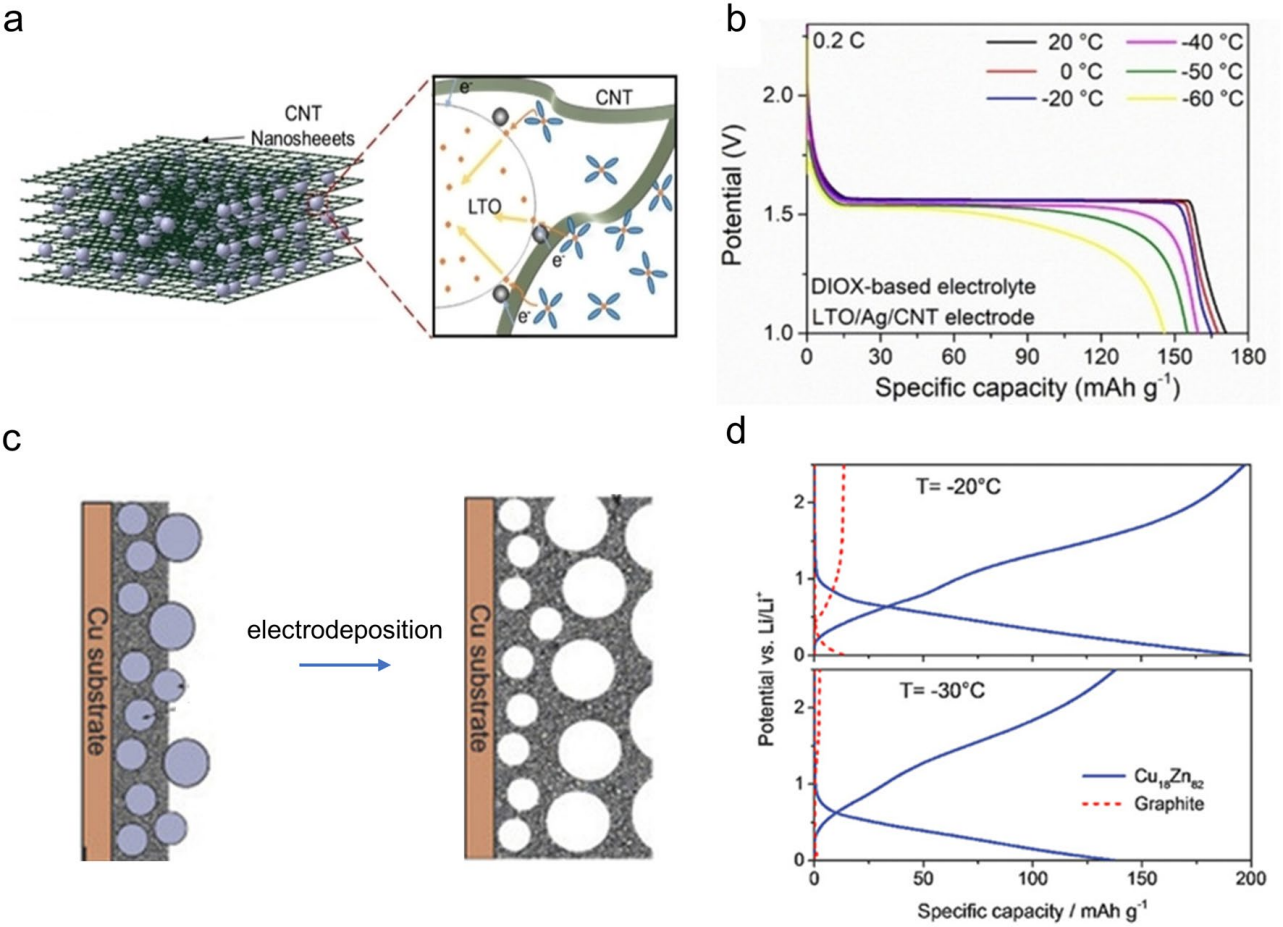
Examples of multiphase systems on anode materials for LT LIBs. **a** Schematic diagram of LTO/CNT and **b** charge voltage profiles over various temperatures. Copyright 2020, Wiley–VCH [122]. **c** Scheme illustration of the synthesis process and **d** the LT performance of porous Cu_20_Zn_80_. Copyright 2017, Wiley–VCH [123]

Composite generally refers to a mixture of multiple materials, among which carbon materials are perhaps the most extensively employed components for composite materials in LIBs. The incorporation of carbon-based materials could significantly improve the electrical conductivity, facilitate the charge transfer processes, and enhance the overall LT performance of composites [126]. For instance, MnO nanoparticles anchored on graphite (MnO@Graphite) were applied as a low-temperature anode [59]. Due to the integrated structure, the MnO@Graphite anode exhibited a capacity of 456 mAh g^−1^ after 320 cycles at − 25 °C. Then, a multiscale (Nb_2_O_5_/TiNb_2_O_7_)@C nanoarchitecture (CTN) was prepared via a simple solvothermal method [121]. The existence of abundant grain boundaries within this composite facilitated the diffusion of Li^+^ and increased the active sites for Li^+^ storage. The CTN anode material exhibited a capacity of 110 mAh g^−1^ after 80 cycles at − 40 °C (0.03 A g^−1^) and 162 mAh g^−1^ after 100 cycles at − 20 °C (0.06 A g^−1^), which equaled 40% and 71% of its RT capacity, respectively. Other carbon materials have also been utilized as active components for composites, including carbon nanotubes [122], hierarchical carbon networks [127], and carbon flakes [128]. As shown in Fig. 7a, b, a composite anode, which consisted of LTO nanoparticles and carbon nanotubes [122], delivered an initial capacity of 140 mAh g^−1^ (0.04 A g^−1^) at − 60 °C. The synergetic effects between them could shorten the ions pathway, reduce the overpotential, and suppress the side reactions, resulting in an improved LT performance.

#### Alloying

Alloys mainly involve the alloyed anode materials among metals, with a primary focus on incorporating metallic elements (e.g., Sn, Pb, Bi) situated within the IVA and VA groups. Constructing alloys with active or inactive metals [129] could enhance electronic conductivity and relieve volume change, resulting in an improved LT performance. For instance, the inclusion of an electrochemically inactive phase could buffer the big volumetric change of Sn during the lithiation and delithiation processes [130]. Then, a 3D porous Cu–Zn alloy [123] was synthesized using a template method (Fig. 7c); the Cu_18_Zn_82_ alloyed anode delivered a stable capacity of 137 mAh g^−1^ (0.1 A g^−1^, Fig. 7d) after 30 cycles at − 30 ℃. Besides, Ma et al. [124] developed nanoporous-structured ternary alloys by selectively etching Al out, which consisted of Cu, Ge, and Al elements (NP-CGA). The NP-CGA anode with lower Al content showed a better Li storage capacity of 122.9 mAh g^−1^ (1 A g^−1^) than the NP-CGA anode with higher Al content (63.6 mAh g^−1^) at − 20 °C.

It is therefore evident that the utilization of the aforementioned modification strategies, including morphology control, microstructural engineering, surface coating & interface modifications, and construction of multi-phased materials, has demonstrated substantial potential for improving the LT performance of anode materials in LIBs. It is noteworthy that these strategies are not isolated alone, but rather interconnected with each other. Generally, it is encouraged to improve the LT performance through the combination and cross-amalgamation of various strategies. For instance, a Cu-doped porous TiNb_2_O_7_ microsphere [53] is proposed by combining structural design (lattice distortions and oxygen vacancies) and morphology regulation (3D material). This modified TiNb_2_O_7_ exhibited a narrow band gap, enhanced electronic conductivity, and rapid Li^+^ diffusivity, leading to a capacity of 76.6 mAh g^−1^ after 200 cycles at − 20 °C.

**Fig. 8:**
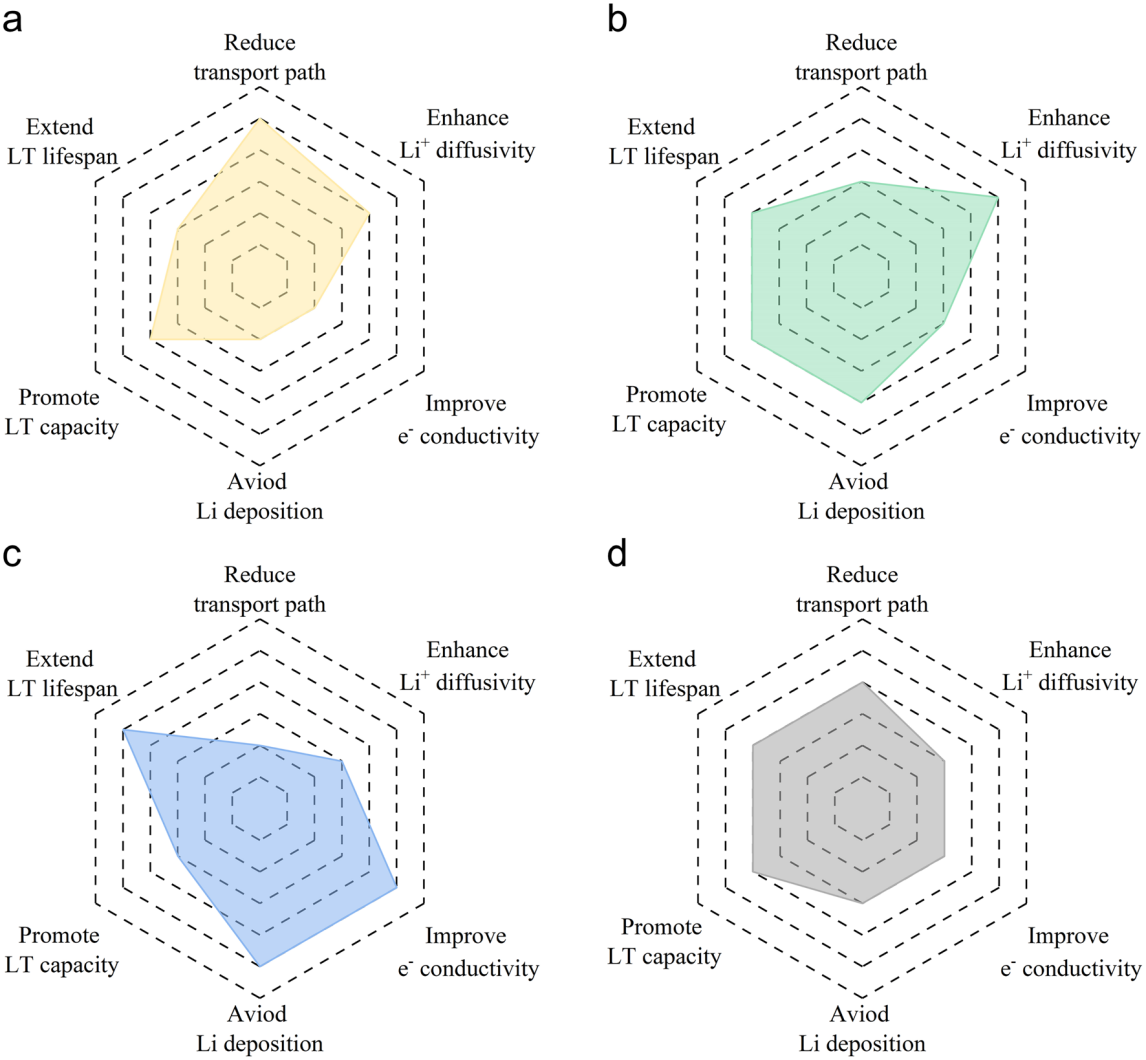
Radar charts of the comparison for different modification strategies: **a** morphology regulation, **b** structural design, **c** surface & interface engineering, and **d** multiphase system

Based on the explanation of theoretical depth and its actual low-temperature performance, Fig. 8 presents a comparative analysis, where a radar chart is employed to assess the merits and demerits of each modification strategy across various dimensions, including the reduction of Li^+^ transport path, enhancement of Li^+^ diffusivity, improvement of electronic conductivity, suppression of lithium deposition, boost of LT capacity, and extension of LT lifespan. An example is the comparison of the effects of each modification strategy in enhancing Li^+^ diffusivity: (a) For the morphology regulation strategy, the diffusion of Li^+^ is promoted indirectly by reducing the diffusion distance and shortening the diffusion time; (b) for the structural design strategy, the Li^+^ diffusivity is directly promoted by increasing the lattice spacing and decreasing the ion diffusion barrier; (c) while a conductive material is coated on the surface of anode, the Li^+^ diffusivity can be facilitated by accelerating the transfer of electrons and lithium ions; (d) the existence of grain boundaries and heterogeneous structures in the multiphase system is conducive to the rapid diffusion of lithium ions. Based on the theoretical depth and practical effect involved in improving Li^+^ diffusivity, it is expected that the usability of each strategy is as follows: structural design > morphology regulation > multiphase system ≈ surface & interface engineering.

Among them, morphology regulation primarily directs its focus toward reducing the ion transport path and promoting Li^+^ diffusivity via adjusting the morphology of nanoparticles, thereby enhancing the LT capacity. Structural design revolves around expediting Li^+^ diffusivity by judiciously adjusting the crystal structure, thus ensuring commendable LT performance. Surface & interface engineering emphasizes the modification of electrode/electrolyte interface properties to attain high electronic conductivity while effectively suppressing the growth of lithium dendrites, thus leading to a marked improvement in LT cycle performance. The multiphase system is a commonly used method that involves the combination of distinct materials to take advantage of their respective advantages, thereby effectively improving the LT performance. It is evident that different modification strategies exhibit diverse emphases, and the combination of these strategies to design innovative LT anodes with superior kinetic properties shows great application potential.

## Summary and Perspectives

**Fig. 9:**
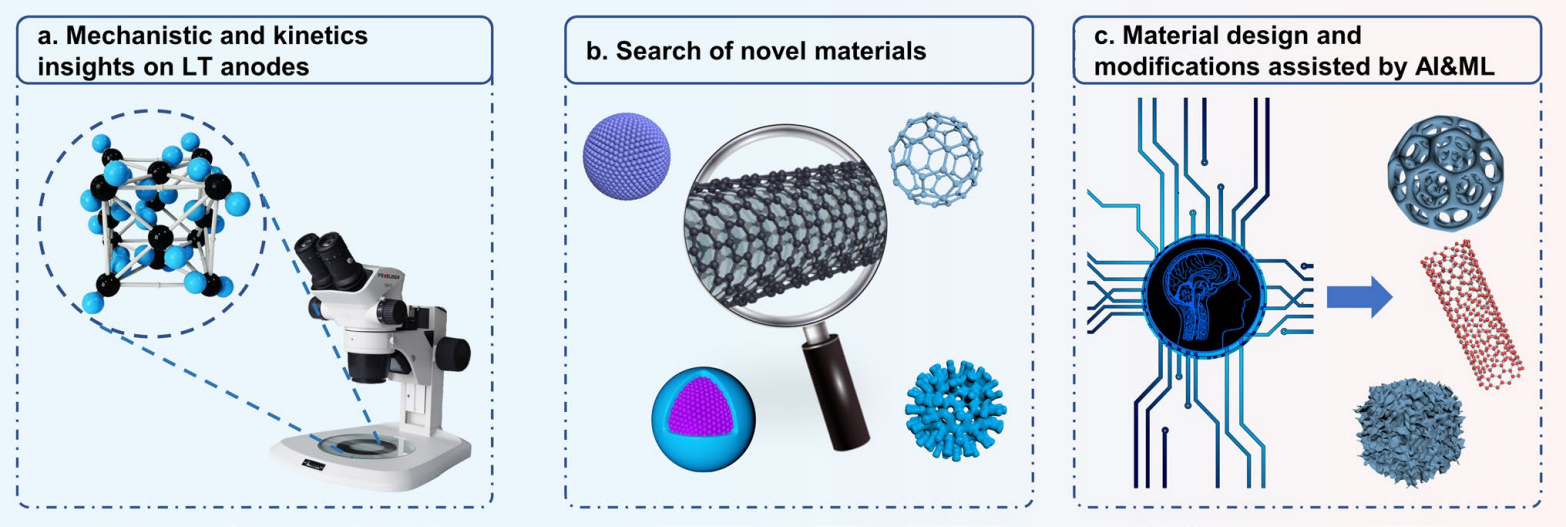
The challenges for the future development of anodes in LT LIBs

In summary, the enhancement of low-temperature LIBs needs to solve several technical limitations, ranging from high electrolyte viscosity, sluggish redox kinetics, large bulk resistance, considerable electrochemical polarization, and inevitable growth of lithium dendrites. These factors significantly impact the available capacity and power density, resulting in unsatisfactory LT performance of LIBs. Among them, achieving fast Li ions diffusion within the anode materials is recognized as a critical factor. Ideally, anode materials should possess high electronic conductivity, rapid ion diffusion, and the capability of inhibiting dendrite formation on the surface. Global scientists have developed a range of LT anode materials and employed a variety of modification strategies, including morphology regulation, structural design, surface & interface engineering, and multiphase system, to adjust their properties. And some achievements have been accomplished in the fabrication of anodes with reduced Li^+^ diffusion distances, enhanced electronic and ionic conductivities, and modified electrolyte–electrode interphases for anode materials in LT LIBs. Yet, it has to be admitted that there are still many challenges remaining to be solved in the development of practical and high-performance LT anodes, which we will briefly discuss in the following sections (see Fig. 9 for the schematic).

### Mechanistic and Kinetics Insights on LT Anodes

An accurate and thorough understanding of temperature-dependent Li^+^ transportation mechanism and kinetics within anodes is of utmost importance for further improving the electrochemical performance. Further insights concerning the detailed information during battery operations (such as the desolvation process of Li^+^ through the interface, the Li^+^ migration behavior, and the mechanism of structural evolution) may be extracted by advanced characterization techniques (i.e., in situ Raman spectroscopy, cryogenic transmission electron microscopy, and neutron diffraction techniques). For instance, with the aid of high-energy electron-beam irradiation in atomic-resolution scanning transmission electron microscopy, the grain boundaries migration process was revealed at the atomic scale [131]. Furthermore, theoretical calculations are also useful in elucidating the fundamental transportation mechanisms of Li^+^. The combination of advanced characterization techniques and in-depth theoretical calculations can effectively uncover the critical factors that affect Li^+^ transportation and facilitate the future development of LT anode materials. For instance, by combining electrochemical models with experimental data, the high responsivity of isolated lithium to battery operations was revealed [132]. This mechanistic insight into the behavior of isolated Li can inspire the future development of LIBs.

### Search of Novel Materials

As mentioned above, many materials (metal oxides, alloys, carbon-based composites, etc.) have been explored for potential high-performance LT anodes. And significant achievements in LT performance for anode materials have been made through various modification strategies, including ion doping, morphology regulation, and structural design. To make further improvements toward satisfying LT performance, it is necessary to search novel electrode materials. For example, very recently, Wadsley-Roth shear structural materials and transition metal sulfides/oxides have shown remarkable potential for next-generation high-rate and high-safety LIBs. For instance, a disordered rock salt (Li_3_V_2_O_5_) was developed as an anode material [133]. Owing to its high-rate intercalation reaction process and structural stability, it exhibited exceptional rate capability (over 40% of its capacity in 20 s) and good cycling performance (negligible capacity decay after 1000 cycles). Besides, a novel mesoporous titanium niobium oxide (Ti_0.88_Nb_0.88_O_4−x_@C) was designed to meet the requirement of low-temperature sodium storage [134]. Due to its fast Na diffusion kinetics, a discharge capacity of 161 mAh g^−1^ was delivered at − 40 °C. Considering the sluggish ion-diffusion kinetics of Na^+^ brought by its larger radius, it is speculated that this novel material may have considerable low-temperature lithium storage performance. Therefore, it is rather essential that future research on LT LIBs keeps the focus on searching of novel electrode materials.

### Material Design and Modifications Assisted by AI&ML

One of the most encouraging advancements for material science in this decade can be the tremendous progress on computational techniques (on both hardware and software), among which artificial intelligence (AI) and machine learning (ML) are expanding their applications toward every scientific domain. specifically, in the field of battery research, AI and ML have also emerged as valuable tools for assisting experimental investigations, predicting material properties, and guiding electrode design/modifications. For instance, an artificial intelligence model was established by applying a database with key parameters [135]. This AI model exhibited sufficient effectiveness in predicting, designing, and producing graphite-based anodes. Upon the gradual establishment of LT electrode material databases, AI&ML is expected to facilitate a deeper understanding of the intricate composition–structure–performance relationships for these materials. This interdisciplinary research method has the potential to inject fresh vitality into the realm of battery research and indicates new opportunities for the advancement of low-temperature LIBs.

Above all, addressing the aforementioned challenges associated with LT batteries requires collaboration across multiple fields. It is crucial to consider all fundamental components and their compatibility in low-temperature environments. With continuous exploration and dedicated research efforts, we hold a strong belief that significant breakthroughs in the field of low-temperature LIBs are feasible and inevitable.
